# Genome-based characterization of two Colombian clinical *Providencia rettgeri* isolates co-harboring NDM-1, VIM-2, and other β-lactamases

**DOI:** 10.1186/s12866-020-02030-z

**Published:** 2020-11-12

**Authors:** Adriana Piza-Buitrago, Verónica Rincón, John Donato, Sandra Yamile Saavedra, Carolina Duarte, Jaime Morero, Laurent Falquet, María Teresa Reguero, Emiliano Barreto-Hernández

**Affiliations:** 1 Bioinformatics Group, Biotechnology Institute, Universidad Nacional de Colombia, Bogotá, Colombia; 2 Molecular Epidemiology Laboratory, Biotechnology Institute, Universidad Nacional de Colombia, Bogotá, Colombia; 3 Grupo de Microbiología, Instituto Nacional de Salud, Bogotá, Colombia; 4 Biochemistry/Bioinformatics Unit, Université de Fribourg and Swiss Institute of Bioinformatics, Fribourg, Switzerland

**Keywords:** Whole-genome sequencing, *bla*_CTX-M-15_, *bla*_CMY-2_, *bla*_OXA-10_, And *bla*_TEM-1_

## Abstract

**Background:**

*Providencia rettgeri* is a nosocomial pathogen associated with urinary tract infections and related to Healthcare-Associated Infection (HAI). In recent years isolates producing New Delhi Metallo-β-lactamase (NDM) and other β-lactamases have been reported that reduce the efficiency of clinical antimicrobial treatments. In this study, we analyzed antibiotic resistance, the presence of resistance genes and the clonal relationship of two *P. rettgeri* isolates obtained from male patients admitted to the same hospital in Bogotá – Colombia, 2015.

**Results:**

Antibiotic susceptibility profile evaluated by the Kirby-Bauer method revealed that both isolates were resistant to third-generation carbapenems and cephalosporins.

Whole-genome sequencing (Illumina HiSeq) followed by SPAdes assembling, Prokka annotation in combination with an in-house Python program and resistance gene detection by ResFinder identified the same six β-lactamase genes in both isolates: *bla*_NDM-1_*, bla*_VIM-2_*, bla*_CTX-M-15_*, bla*_OXA-10_*, bla*_CMY-2_ and *bla*_TEM-1_. Additionally, various resistance genes associated with antibiotic target alteration (*arn*A, *Pmr*E, *Pmr*F, *Lpx*A, *Lpx*C, *gyr*B, *fol*P, *mur*A, *rpo*B, *rps*L, *tet*34) were found and four efflux pumps (*Ros*AB, *Emr*D, *mdt*H and *cml*A).

The additional resistance to gentamicin in one of the two isolates could be explained by a detected SNP in CpxA (Cys191Arg) which is involved in the stress response of the bacterial envelope.

Genome BLAST comparison using CGView, the ANI value (99.99%) and the pangenome (using Roary) phylogenetic tree (same clade, small distance) showed high similarity between the isolates. The rMLST analysis indicated that both isolates were typed as rST-61,696, same as the RB151 isolate previously isolated in Bucaramanga, Colombia, 2013, and the FDAARGOS_330 isolate isolated in the USA, 2015.

**Conclusions:**

We report the coexistence of the carbapenemase genes *bla*_NDM-1_, and *bla*_VIM-2_, together with the β-lactamase genes *bla*_CTX-M-15_*, bla*_OXA-10_*, bla*_CMY-2_ and *bla*_TEM-1_, in *P. rettgeri* isolates from two patients in Colombia. Whole-genome sequence analysis indicated a circulation *of P. rettgeri* rST-61,696 strains in America that needs to be investigated further.

**Supplementary Information:**

The online version contains supplementary material available at 10.1186/s12866-020-02030-z.

## Background

*Providencia rettgeri* is an opportunistic pathogen mainly associated with urinary tract infections [1]; it can be a causative agent of bacteremia [2], traveler’s diarrhea [3], eye infections [4] and wounds [5] and in a low incidence, of meningitis [6] and pyelonephritis [7] . In recent years, *P. rettgeri* has taken on major importance due to the emergence of carrier isolates of the New Delhi metallo-β-lactamase NDM-1, a carbapenemase that hydrolyzes all β-lactams for clinical use except aztreonam [8, 9]. NDM-1 was first described in 2009, in a *Klebsiella pneumoniae* isolated in a Swedish hospital from a patient who had been previously hospitalized in India [10]. Since then, *P. rettgeri* isolates producing NDM-1 have been reported in different parts of the world, including Asia (Nepal, Israel, India, Korea, China, Pakistan) [11–16] South America (Brazil, Argentina, Colombia, Ecuador) [17–21] North America (Canada, Mexico) [22, 23] South Africa (Nigeria) [24] and South East Europe (Bulgaria) [25].

The first report of *P. rettgeri* producing NDM-1 in Colombia was published in 2015. The isolates were obtained in 2012–2013, in the departments of Santander and Cundinamarca, and in the capital city, Bogotá [20]. In 2016, the Colombian Antimicrobial Resistance Surveillance Program in Healthcare-Associated Infections (HAI) reported *P. rettgeri* as one of the most commonly sent of the order Enterobacterales for confirmation of carbapenemases [26], and a study conducted by the National Institute of Health (INS) of Colombia in 2018 (unpublished data) *P. rettgeri* was determined to be the second most common carrier of the *bla*_NDM-1_ gene among Enterobacteriaceae in this country.

The presence of *bla*_NDM-1_ in a bacterial isolate is a challenge for clinical therapeutics. However, it is a greater challenge when it is co-harboring with other resistance determinants because it results in a greater limitation of the treatment options [27–29]. Worldwide, few reports of *P. rettgeri* notifying coexistence of *bla*_NDM-1_ with other resistance determinants have been published. In 2014, the first coexistence of metallo-β-lactamase *bla*_NDM-1_ was reported along with *armA* encoding a 16S rRNA methylase in clinical isolates of *P. rettgeri* in Nepal [11]. Recently, the coexistence of *bla*_NDM-1_, along with other resistance determinants such as *bla*_PER-1_-type extended-spectrum β-lactamases (ESBLs) in Korea [14], and class D and A β-lactamases *bla*_OXA-48_ and *bla*_TEM-1_, respectively, in Turkey [30] have been described. Similarly, other coexistence in *P. rettgeri* that do not include the *bla*_NDM-1_ gene have been described. In 2007, the co-production of PER-1, VIM-2 and *Arm*A in *Providencia spp*. isolates in Korea was reported [31]; and in Nigeria, an isolation of *P. rettgeri* was reported in 2011 carrying genes *bla*_*O*XA-10_, *bla*_VEB-1_, *bla*_*C*MY-4_ and *bla*_TEM-1_ [32].

In this study, we used whole-genome sequencing (WGS) for characterization of two *P. rettgeri* isolates that carried *bla*_NDM-1_ and *bla*_VIM-2_ carbapenemases, recovered in March and December 2015 at a fourth-level health care institution in Colombia.

## Results

### Amplification and sequencing of 16S rRNA

The 16S rRNAs obtained by PCR and sequenced for both isolates (1490 pb each of them), were aligned against the NCBI DNA nr database using blastn. The best hits of isolates GMR-RA257 and GMR-RA1153, corresponded to the seqref 16S RNA of *P. rettgeri* strain RB-151 (coverage 100%, value E 0.0, identity 100%) and *P. rettgeri* strain FDAARGOS_330 (coverage 100%, value E 0.0, identity 99%), which confirmed their species identity as *Providencia rettgeri.*

### Antibiotic resistance of *P. rettgeri* isolates

The isolates GMR-RA257 and GMR-RA1153 presented a similar susceptibility profile. Both isolates were resistant to carbapenems (imipinem, meropenem), second- (cefoxitin) and third-generation cephalosporins (ceftazidime, cefotaxime, cefepime), amikacin and trimethoprim/sulfamethoxazole and were susceptible to aztreonam and piperacillin/tazobactam.

Some differences in the phenotypic profile of susceptibility were observed: GMR-RA257 showed an intermediate susceptibility to gentamicin, while GMR-RA1153 showed intermediate susceptibility to ertapenem. The phenotypic susceptibility profile of the *P. rettgeri* isolates showed resistance to three or more families of antibiotics, and therefore, both were classified as multi-drug resistant (MDR) according to standardized international terminology [33].

The imipenem-EDTA/SMA double-disk synergy test confirmed the production of enzymes of the metallo-β-lactamase (MBL) type. However, the modified Hodge test (MHT) was negative and the 3-Aminophenylboronic acid (APB)-based disk/microdilution test did not detect the production of serine-type β-lactamases.

### Assembly and annotation of GMR-RA257 and GMR-RA1153 genomic sequences

Whole genome sequencing (WGS) of GMR-RA257 and GMR-RA1153 yielded 20,13 and 5,93 millions of reads respectively. These results were obtained using Illumina HiSeq 2500, and quality control was provided using FastQC, obtaining a high-quality score of Q30 (results not shown). The G + C content for these isolates was 40.5%, considered common for this species [9, 34, 35]. Detailed sequencing characteristics of GMR-RA257 and GMR-RA1153 can be found in Table S1. The estimated size of GMR-RA257 and GMR-RA1153 were 4,84 Mb for both isolates, with a greater coverage at a depth of 744X and 216X, respectively. A total of 71 tRNAs, 7 rRNAs and 4452 coding sequence (CDS) were annotated in both genomes. Those values are in the range of the previously reported genomes of *P. rettgeri* [9, 36]. The whole-genome sequences of *P. rettgeri* GMR-RA257 and GMR-RA1153 were deposited in the DDBJ/ENA/GenBank databases under the accession numbers VRPG00000000 and VRPH00000000 respectively. Details of the assembly and annotation for *Providencia rettgeri* isolates GMR-RA257 and GMR-RA1153 can be found in the supplementary Table S1.

The GMR-RA1153 and GMR-RA257 antimicrobial resistomes were obtained from their GFF files produced by Prokka v1.13. The resistomes included genes three different antibiotic resistance categories: antibiotic degradation or modification; antibiotic target alteration; and efflux pumps (Table 1). There was no difference in the type and the numbers of resistance genes present in both isolates: they showed a 100% identity with genes previously reported in the GenBank. Only the *CpxA* gene (histidine kinase sensor of the two-component system Cpx-TCS, involved in the stress response of the bacterial envelope) of GMR-RA1153 had a 99.93% identity with genes previously reported in GenBank and its alignment with the *CpxA* gene of GMR-RA257 showed a change in one amino acid (Cys191Arg).

**Table 1:** Resistance-associated genes in *Providencia rettgeri* strains GMR-RA257 and GMR-RA1153

Resistance category		Resistance gene		Groups/Classes	Gene location (C) Chromosome or (P) Plasmid ^e^	GMR-RA257 ^**a**^	GMR-RA1153 ^**a**^
**Antibiotic degradation or modification**		*bla*_NDM-1_		β-lactams	P	100.00	100.00
		*bla*_VIM-2_			P	100.00	100.00
		*bla*_CTX-M-15_			P	100.00	100.00
		*bla*_CMY-2_			P	100.00	100.00
		*bla*_OXA-10_			P	100.00	100.00
		*bla*_TEM-1_			P	100.00	100.00
		*ant*(2″)-Ia		Aminoglycosides	P	100.00	100.00
		*aph(*3′)-VI			P	100.00	100.00
		*aac*(6′)-Il			P	100.00	100.00
		*aad*A1			P	100.00	100.00
		*sul*1		Sulphonamides	P	100.00	100.00
		*sul*2			P	100.00	100.00
		*qnrD1*		Fluoroquinolones	P	100.00	100.00
		*cml*A1		Phenicol	C	100.00	100.00
		ARR-2		Rifampicin	P	100.00	100.00
		*tet* (59)		Tetracyclines	C	100.00	100.00
		*dfr*A6		Trimethoprim	P	100.00	100.00
**Antibiotic target alteration**		*arn*A		Peptide antibiotics	C	100.00	100.00
		*Pmr*E			C	100.00	100.00
		*Pmr*F			C	100.00	100.00
		*Lpx*A			C	100.00	100.00
		*Lpx*C			C	100.00	100.00
		*gyr*B^b^		Fluoroquinolone antibiotics	C	100.00	100.00
		*par*C^b^ *parE*^b^			P P	100.00 100.00	100.00 100.00
		*fol*P		Sulphonamides	C	100.00	100.00
		*mur*A		Fosfomycin	C	100.00	100.00
		*rpo*B		Rifamycin	C	100.00	100.00
		*rps*L		Aminoglycoside antibiotics	C	100.00	100.00
		*tet*34		tetracyclines	C	100.00	100.00
**Efflux pumps**	MdtABC-TolC	RND^c^	*mdt*A	Aminocoumarin antibiotics	C	100.00	100.00
			*mdt*B		C	100.00	100.00
			*mdt*C		C	100.00	100.00
			*bae*R		C	100.00	100.00
			*bae*S		C	100.00	100.00
			*Cpx*A		C	99.93	99.93
			*Cpx*R		C	100.00	100.00
			*Tol*C		C	100.00	100.00
	EmrAB-TolC	MFS^d^	*emr*A	Fluoroquinolones	C	100.00	100.00
			*emr*B		C	100.00	100.00
			*emr*R		C	100.00	100.00
			*Tol*C		C	100.00	100.00
	AcrAB-TolC	RND^b^	*Acr*A	Tetracyclines, cephalosporins, phenicol, rifamycin, fluoroquinolones	C	100.00	100.00
			*Acr*B		C	100.00	100.00
			*Acr*R		C	100.00	100.00
			*Tol*C		C	100.00	100.00
	RosAB	MFS^d^	*ros*A	peptides	C	100.00	100.00
			r*os*B		C	100.00	100.00
	EmrD	MFS^d^	*Emr*D	antibiotic efflux	C	100.00	100.00
	mdtH	MFS^d^	*mdt*H	antibiotic efflux	C	100.00	100.00
	cmlA5	MFS^d^	*cml*A5	phenicol antibiotic	P	100.00	100.00

^a^ % identity with GenBank RefSeq DNA sequences

^b^ Gene genetic variants described previously causing resistance to fluoroquinolones [37, 38]

^c^ RND: resistance-nodulation-cell division (RND) antibiotic efflux pump

^d^ MFS: major facilitator superfamily, antibiotic efflux pump

^e^ The gene plasmid location was confirmed by the high deep of coverage of their contigs in comparison with the mean coverage of the genome contigs, and the plasmid contig identification made using PlasmiSPAdes (SPAdes v3.8.0) [39] and PlasFlow 1.1 [40]

The further results showed that plasmid-mediated genes associated with resistance to β-lactam antibiotics were the most abundant in the antibiotic degradation or modification category. This includes MBL genes encoding *bla*_NDM-1_ and *bla*_VIM-2,_ the ESBL gene *bla*_CTX-M-15_, the AmpC gene *bla*_CMY-2_, and other β-lactamases genes like *bla*_OXA-10_, and *bla*_TEM-1_. Further commonly detected genes were associated with aminoglycoside resistance (*ant*(2″)-Ia, *aph(*3′)-VI, *aac*(6′)-Il, and *aad*A1), and sulfonamide resistance (*sul1* and *sul2*) (Table 1).

The *arnA, PmrE, PmrF, LpxA, and LpxC* genes, associated with resistance to peptide antibiotics (bacitracin, colistin and polymyxin B) were the most abundant in the antibiotic target alteration category. Additionally, six different efflux pumps were identified, belonging to the major facilitator superfamily (MFS) and represented by pumps of the resistance-nodulation-cell division (RND) family (Table 1).

The carbapenemase genes *bla*_NDM-1_ and *bla*_VIM-2_ were found in two different contigs. Within the *bla*_NDM-1_ contig (7794 pb) was present the IS*91* family transposase gene that could increase the *bla*_NDM-1_ expression level [41, 42]. This contig had 100% identity and 100% coverage with bigger plasmids present in many other bacteria like in *Escherichia coli* (GI: MN604268.1), *Acinetobacter baumannii* (GI: CP027528.1) and *P. rettgeri* strain RB151(GI: CP017672.1). The *bla*_VIM-2_ contig (2011 pb) had 100% identity and coverage within 68 and 79% with bigger plasmids present in other bacteria like in *Pseudomona aeruginosa* (GI: KR337992.1), *Acinetobacter baumannii* (GI: AF324464.1) and *Pseudomona putida* (GI: AF327064.1). Within the small *bla*_VIM-2_ contig was present the gene class 1 integron integrase IntI1 associated with antimicrobial resistance gene cassette. The plasmid location of the *bla*_VIM-2_ and the other plasmid resistance-associated genes (Table 1) was supported too by the high deep of coverage of their contigs in comparison with the mean coverage of the genome contigs, and the plasmid contig identification made using PlasmiSPAdes (SPAdes v3.8.0) [39] and PlasFlow 1.1 [40].

### Ribosomal multilocus sequence typing (rMLST)

The rMLST was done using the PubMLST website https://pubmlst.org/rmlst/ for the genomes of the GMR-RA257 and GMR-RA1153 study and 16 *Providencia rettgeri* genomes available in the GenBank database. Ten of the 18 *P. rettgeri* genomes evaluated were assigned to seven different rST types, as follows: rST-61,696 was the predominant rST corresponding to the genomes of the study GMR-RA257, GMR-RA1153 and two genomes: RB151 isolated in Bucaramanga, Colombia, in 2013 and FDAARGOS_330 isolated in Washington D.C., the United States in 2015, both of them recovered from urine samples. The rST-37,417, rST-37,410, rST-63,059, rST-63,070, rST-37,423 and rST-37,424 were assigned to an individual reference’s genomes (Table 2).

**Table 2:** rST and β-lactamase presence in GMR-RA257, GMR-RA1153 and 16 Providencia *rettgeri* genomes previously reported

Isolated	AC DDBJ/ENA/GenBank	Country	Year	rST	β-lactamases
GMR-RA257	VRPG00000000	Colombia	2015	61,696	*bla*_NDM-1_ *bla*_OXA-10_ *bla*_CMY-2_ *bla*_CTX-M-15_ *bla*_TEM-1_ *bla*_VIM-2_
GMR-RA1153	VRPH00000000	Colombia	2015	61,696	*bla*_NDM-1_ *bla*_OXA-10_ *bla*_CMY-2_ *bla*_CTX-M-15_ *bla*_TEM-1_ *bla*_VIM-2_
RB151	NZ_CP017671	Colombia	2013	61,696	*bla*_NDM-1_ *bla*_OXA-2_ *bla*_TEM-1_
FDAARGOS_330	NZ_CP027418	USA	2015	61,696	N/A
Dmel1	NZ_CM001774	USA	2012	37,417	N/A
DSM1131	ACCI00000000	USA	2009	37,410	N/A
AR_0082	NZ_CP029736	USA	2015	NA	*bla*_NDM-1_
UBA5024	DICO00000000	USA	2015	NA	N/A
PR1	NOWC00000000	USA	2016	63,059	*bla*_PER-7_ *bla*_IMP-27_ *bla*_IMP-64_ *bla*_IMP-67_
CCBH1880	JSEQ00000000	Brazil	2014	37,423	*bla*_NDM-1_ *bla*_OXA-10_
729–12	LYBX00000000	Brazil	2012	NA	N/A
PR002	NXKD00000000	South Africa	2013	63,070	*bla*_OX1–1_ *bla*_OXA-2_ *bla*_OXA-10_ *bla*_PER-7_
H1736	CVLT00000000	Israel	2011	37,424	*bla*_NDM-1_ *bla*_OXA-10_
MR4	LCVM00000000	India	2013	NA	N/A
TUM9994	BGMI00000000	Japon	2010	NA	*bla*_CTX-M-62_
Pret_2032	QKNQ00000000	Spain	2015	NA	*bla*_NDM-1_ *bla*_CARB-2_ *bla*_FOX-5_ *bla*_OXA-1_ *bla*_VIM-1_
NCTC11801	UGTZ00000000	United Kingdom	Strain Collection	NA	N/A
NCTC7476	UAUG00000000	United Kingdom	Strain Collection	NA	N/A

*NA* Not assigned rST because the ribosomal gene alleles in the genome have not been previously reported to the BIGSdb platform

*N/A* not available

### Whole genome comparisons

The pangenome analysis carried out by Roary identified a total of 12, 539 genes, including 1, 217 (~ 10%) for the core genome, in the 18 genomes used in this work. The 11,322 non-central genes were divided into 6980 accessory genes and 4342 unique genes.

**Fig. 1:**
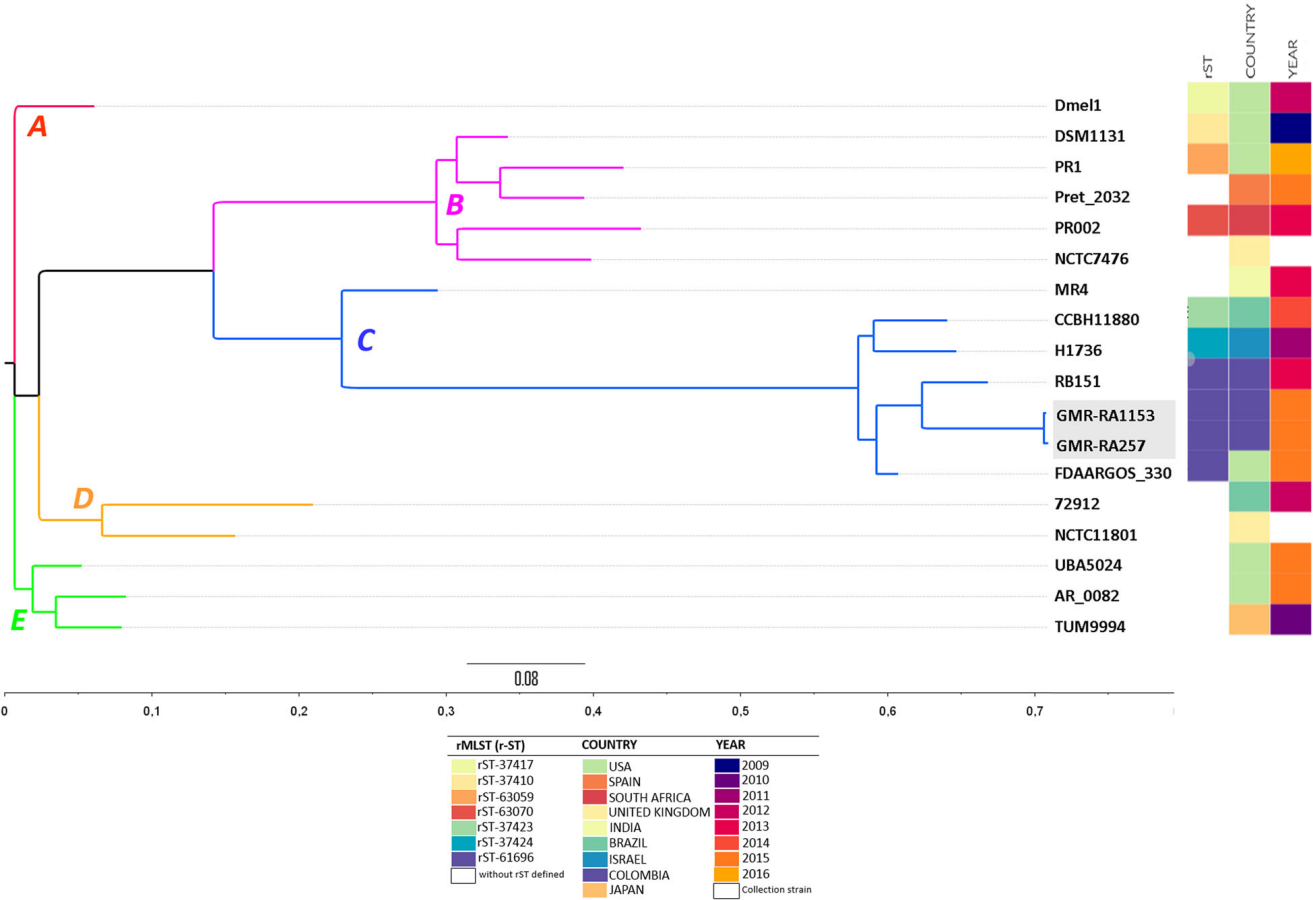
Pangenome phylogenetic tree of the GMR-RA257, GMR-RA1153 and 16 Providencia *rettgeri* genomes previously reported

A phylogenetic tree was built from the core and accessory genes presence/absence information produced by Roary, using the isolates and the GenBank genomes above described, along with the rST sequence type, the country and year of collection (Fig. 1). Five important clades were observed, where the different rST types were grouped as follows: rST-37,417 (clade A); rST-37,410, rST-63,059, rST-63,070 and two genomes with no rST known (clade B); rST-37,423, rST-37,424, rST-61,696 and one genome with no rST known (clade C); two genomes with no rST known (clade D); and three genomes with no rST known (clade E) (Fig. 1). The phylogenetic tree shows a possible clonal relationship between GMR-RA257 and GMR-RA1153 which were assigned to clade C; both isolates were recovered in Bogotá and were isolated from two patients in the same fourth level hospital in two different times (March and December, respectively). This may indicate that a clone could be circulating within this hospital during that period of time.

The average nucleotide identity (ANI) was calculated using JSpecies. JSpecies provided a 99.99% ANI between GMR-RA257 and GMR-RA1153. Likewise, the ANI value between these genomes and the Colombian reference genome RB151 provided a value ANI of 99.67%, these results showed a close relationship between the Colombian genomes.

**Fig. 2:**
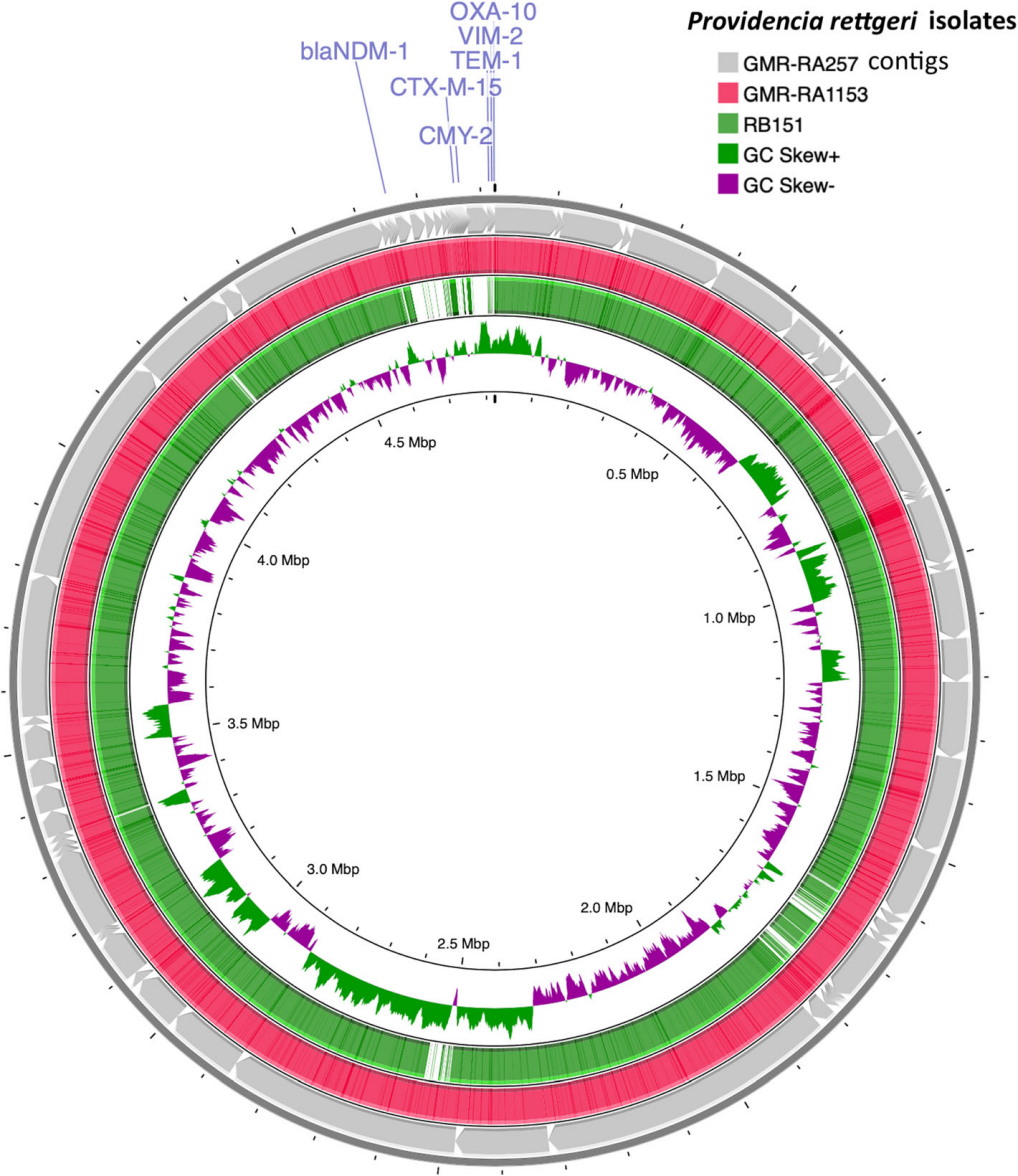
BLAST comparison between GMR-RA257, GMR-RA1153 and *P. rettgeri* RB151 as reference genome. The innermost rings indicate GC skew (purple/green). Rings indicate BLAST identity, from inside to out: Ring 1: RB151 reference genome, Ring 2: BLAST comparison with strain GMR-RA1153, Ring 3: BLAST comparison with the contigs of the strain GMR-RA1153. *bla* plasmid genes are showed in blue

In addition to that, a circular comparison, between the genomic sequences of GMR-RA257 and GMR-RA1153 with the Colombian genome RB151, was performed using CGView Comparison Tool (Fig. 2). The results showed that the three Colombian samples were very similar; most of the compared genomic regions of GMR-RA257 and GMR-RA1153 showed a high genomic similarity (78.1%) with RB151 (Fig. 2).

**Fig. 3:**
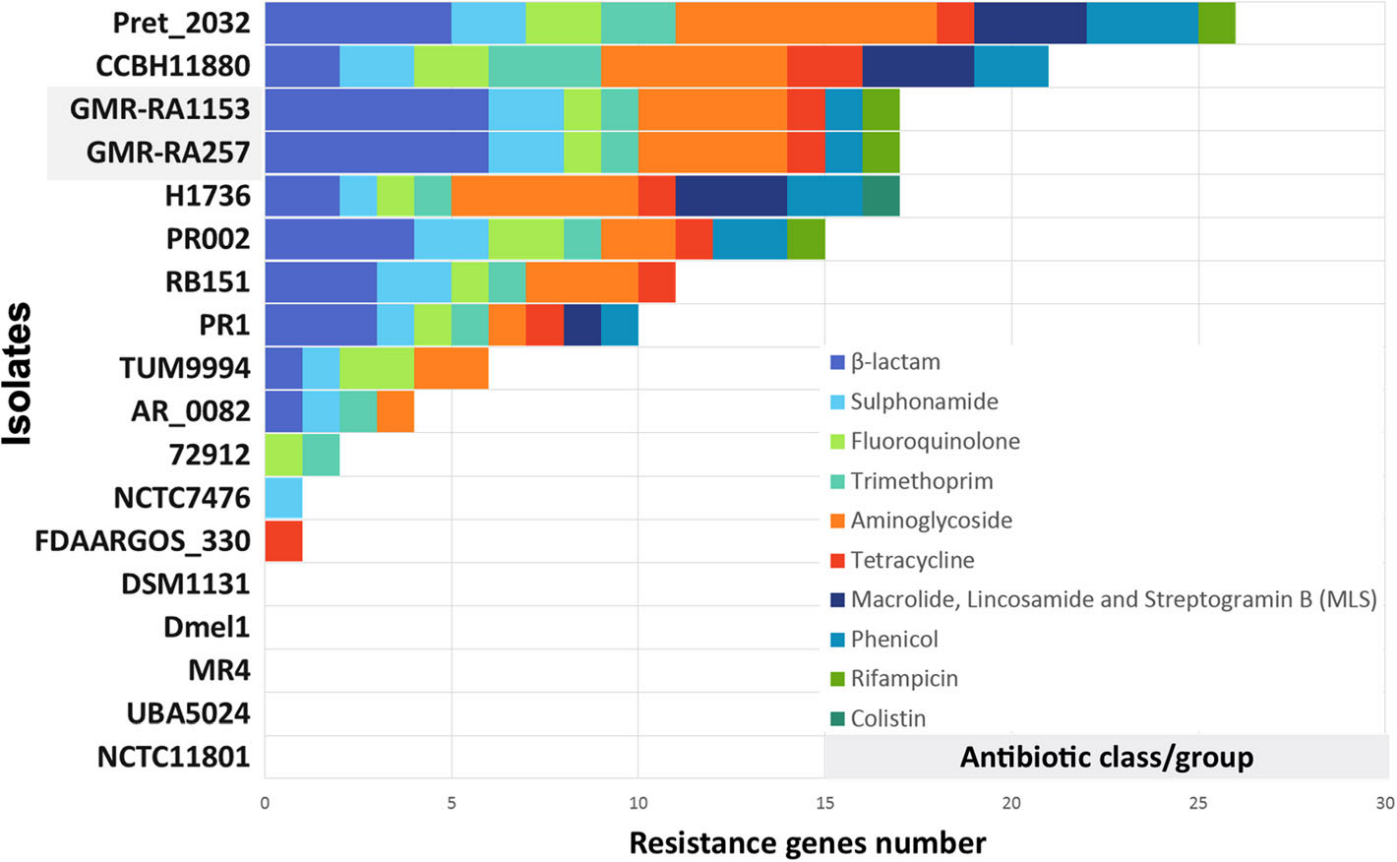
Resistance genes content comparison between GMR-RA257, GMR-RA1153 and 16 Providencia *rettgeri* genomes previously reported

As result of the Resfinder analysis of the 18 genomes of *P. rettgeri*, the genomes pret_2032 and CCBH11880 have the highest number of genomic elements associated with resistance to different categories of antibiotics (Fig. 3). GMR-RA1153 and GMR-RA257 also have several resistance genes associated with eight different categories of antibiotics (results similar to those found in the Prokka annotation), but when compared with the other genomes, they showed the highest number of β-lactamases genes associated with resistance to β-lactam antibiotics (Fig. 3). The comparison of the GMR-RA257 and GMR-RA1153 genomes with the genome of *P. rettgeri* RB151, isolated two years earlier in Colombia, revealed that the three isolates share six different genes associated with resistance: *bla*_NDM-1_, *bla*_TEM-1_, *sul*1, *sul*2, *dfr*A6, *tet* [43]. However, in GMR-RA257 and GMR-RA1153 additional genes were detected that were not present in RB151: *bla*_OXA-10_, *bla*_CMY-2_, *bla*_CTX-M-15_, *bla*_VIM-2_, *qnr*D1, *aad*A1, *aph* (3′)-VI, *aac* (6′)-Il, *cml*A1, *ARR*-2. The detailed results of all the antibiotic resistance genes in each class of antibiotics of each reference genome and collection strains are included in additional Table S2.

We focused our analysis on identifying non-synonymous single nucleotide polymorphisms (nsSNPs) that result in amino acid changes within the functional gene sequences in GMR-RA257 and GMR-RA1153. We established 108 non-synonymous single nucleotide variants (nsSNVs) for GMR-RA257 and GMR-RA1153 using SnpEff. From those, 107 variants were located in genes encoding hypothetical proteins. Only one variant (Cys191Arg) present in the antibiotic resistance gene *CpxA* could explain the increased resistance of GMR-RA1153 to the aminoglycoside antibiotic gentamicin (Table 1).

## Discussion

In this study, we used WGS data to provide information on two isolates of *Providencia rettgeri*. We detected an uncommon accumulation of various β-lactamases genes (*bla*_CTX-M-15_, *bla*_CMY-2_, *bla*_OXA-10_, and *bla*_TEM-1_) genes including carbapenemase genes *bla*_NDM-1_ and *bla*_VIM-2_. To the best of our knowledge this combination of β-lactamases has not been reported before in *Providencia spp*. The isolates showed a similar resistance profile, were resistant to most of the antibiotics evaluated, except aztreonam and piperacillin/tazobactam.

Reports in America [17–19, 21–23] as well as reports in Asia [11–16] and in South Africa [24], commonly associate *P. rettgeri* with high resistance rates to β-lactamic antibiotics, a characteristic that was conserved in Colombian isolates GMR-RA257 and GMR-RA1153. This resistance trait in *P. rettgeri* is frequently associated with the production of metallo-β-lactamase (*bla*_NDM-1_). GMR-RA257 and GMR-RA1153 were susceptible to aztreonam, similarly as previously reported in Israel, Argentina and in a former Colombian report [12, 19, 20]. It is interesting that the presence of *bla*_CTX-M-15_ gene in both isolates did not result in resistance to aztreonam, possibly due to a low expression of this gene. This agrees with different studies where many CTX-M-type ESBL-producing isolates that would previously have been reported as resistant to cephalosporins and aztreonam were now reported as susceptible to these drugs [41, 42, 44]. In isolates GMR-RA257 and GMR-RA1153 the *bla*_CTX-M-15_ was located in a small contig in both isolated (2013 bp and 1924 bp) flanked upstream by a cupin-fold metalloprotein *Wbu*C gene and downstream, by a fragment of transposase gene. Both *bla*_CTX-M-15_ contigs had 92% coverage and 100% identity with bigger plasmids present in different bacteria like *E. coli* (GI: MN158990.1) and *K. pneunoniae* (GI: CP050364.1), and therefore, they could be part of a plasmid ensembled by SPAdes in many contigs, not located into the same scaffold. However, due to the small contig size no assessment of regulatory elements of *bla*_CTX-M-15_ could be made for the two study isolates.

The rMLST analysis of GMR-RA257 and GMR-RA1153 and 16 previously reported genomes, allows to identify seven different rST for ten genomes analyzed. GMR-RA257 and GMR-RA1153 belong to rST-61,696, which is the most predominant rST among the genomes used in this work. An interesting finding was that the genomes GMR-RA257 and GMR-RA1153 obtained in Bogotá in the same hospital, shared the same rST-61,696 and the same clade C in the pangenome phylogenetic tree (Fig. 1), with the Colombian genome RB151 previously reported in Bucaramanga, Colombia, 2013 [8]. This indicate the presence of rST-61,696 in two different geographic locations of the country and the possible wider circulation of this rST in Colombia. Likewise, the FDAARGOS_330 collected in 2015, in the Children’s National Hospital (USA), showed to be closely related to the Colombian genomes GMR-RA257, GMR-RA1153 and RB151 (Fig. 1) and was also typed as rST-61,696. This suggest a wider presence of *P. rettgeri* rST61696 in America.

The pangenome phylogenetic analysis suggests that GMR-RA257 and GMR-RA1153 were clonal considering that they were clustered very closely (distance < 0.01) in the tree (Fig. 1). It was also supported because they came from the same hospital, the ANI was 99.99% and the high identity between them showed by the BLAST comparative circular visualization (Fig. 2). Furthermore, both isolates carried the same resistance genes (Table 1). There were few differences between them and the Colombian *P. rettgeri* RB151 previously reported [8]. These differences were principally in plasmid contigs since they were in the region (4.6–4.9 Mb) where the plasmid contig of the reference genome *P. rettgeri* RB151 was placed (Fig. 2).

As result of the SNP analysis we detected 108 variants of a single non-synonymous nucleotide (nsSNVs). Among those 107 nsSNVs were located in genes encoding hypothetical proteins and only one variant (Cys191Arg) of the GMR-RA1153 genome was located in the *Cpx*A protein. *Cpx*A is a transmembrane histidine kinase sensor of the two-component system Cpx-TCS [45, 46], involved in the stress response of the bacterial envelope by hostile factors such as physical stress (osmolarity), chemical stress (ethanol, pH, detergents) and misfolded proteins [45, 47]. Several studies have implicated the Cpx system in multidrug resistance (MDR), in particular to β-lactam antibiotics (imipenem, cefepime, ceftriaxone, ceftazidime, cefotaxime), chloramphenicol [45] and aminoglycosides [48]. The mutation (Cys191Arg) in *cpx*A is located in the protein region 185–222, where have been reported mutations affecting its activity [49], which could explain the resistance profile differences: resistance and intermediate resistance to gentamicin and intermediate and resistance to ertapenem for GMR-RA1153 and GMR-RA257 respectively. The Kirby-Bauer disk diffusion susceptibility test results interpreted using the 2015 CLSI (Clinical and Laboratory Standards Institute) guidelines [50] showed that this mutation had no influence on resistance to amikacin because both isolates were resistant. Probably, some of the other SNPs could contribute to this variation, but more research will be necessary for understanding how the SNPs located in unknown proteins or in non-coding regions influence the resistance.

The isolates GMR-RA257 and GMR-RA1153 showed an uncommon high number of β-lactamase genes. The ESBL and AmpC β-lactamase genes *bla*_CTX-M-15_ and *bla*_CMY-2_ have not been reported previously in *P. rettgeri* isolates and only one study has described the presence of carbapenemase NDM-1 together with an ESBL as PER-1 [31]. Furthermore, there is not a previously report of NDM-1 together with another ESBL such as CTX-M-15.

Apart from the high number of β-lactamase genes in both study isolates further resistance genes *arnA, PmrE, PmrF, LpxA, LpxC, gyrB, folP, murA, rpoB, rpsL, and tet34* genes were found that have been reported previously in Enterobacterales species but no in *P. rettgeri*. Additionally, four efflux pumps of the MFS family: *RosAB* (*rosA, rosB*), *EmrD*, *mdtH* and *cmlA*, were not previously reported in *P. rettgeri*.

GMR-RA257 and GMR-RA1153 were resistant to the carbapenems imipenem and meropenem; this was highly probable caused by the production of MBL types NDM-1 and VIM-2 [51, 52] and the expression of *bla*_OXA-10_ encoding an enzyme that is currently associated with weak hydrolysis of carbapenem, and whose inclusion is proposed within the group of carbapenem-hydrolyzing class D β-lactamases (CHDLs) [53, 54]. In a study in Nigeria in 2011 a carbapenem susceptible *P. rettgeri* isolate was reported that carried *bla*_OXA-10_, *bla*_VEB-1_ and *bla*_CMY-4_ genes with no presence of the *bla*_NDM-1_ gene [32]. These results show that the presence of MBL as *bla*_NDM-1_ and *bla*_VIM-2_ contribute to carbapenem resistance, which is a serious problem for clinicians, as these are considered last-resort antibiotics. Unfortunately, antimicrobials such as colistin and tigecycline –which are now used as antibiotics to treat multidrug-resistant microorganisms producing carbapenemase [55]– are not a treatment option for *P. rettgeri* isolates, due to the natural resistance of the latter to the said antibiotics [56, 57]. However, a possible treatment option is a combination: Avibactam, non-β-lactam inhibitor with activity against serine β-lactamase classes A, C and D [58]; and aztreonam, resistant to the hydrolysis of MBL. In vitro studies demonstrated good results in the values of minimum inhibitory concentration (MIC) for Enterobacterales isolates that produced MBL and serine β-lactamases such as ESBL and / or AmpC [43, 59]. Aztreonam-avibactam is not yet approved by the U.S. Food and Drug Administration (FDA), but clinicians can administer this combination [43, 59, 60].

## Conclusions

Whole genome analysis of the two clinical P. rettgeri isolates GMB-RA257 and GMB-RA1153 revealed that both isolates harbored an uncommon combination of various β-lactamase genes: *bla*_NDM-1_, *bla*_VIM-2_, *bla*_CTX-M-15_, *bla*_CMY-2_, *bla*_OXA-10_, and *bla*_TEM-1_, that have not been reported previously in this microorganism.

The comparative genomic analyses revealed a close relationship of the isolates; the assigned rST-61,696 was found previously in Colombia and the USA.

The differences of both isolates in gentamicin susceptibility could be explained by a Cys191Arg substitution in the CpxA protein.

Our finding shows the need of detailed and continuous molecular epidemiological surveillance of *P. rettgeri* to assess the regional and worldwide spread of clones and to detect early the acquisition of resistance genes that limit dramatically limit the treatment options for infections with this pathogen.

## Methods

### Bacterial isolates

The Microbiology Group of the National Institute of Health (INS) of Colombia received the isolates GMR-RA257 and GMR-RA1153 that exhibited a decreased susceptibility to carbapenems, as part of the Healthcare-Associated Infection (HAI) Surveillance Program. The isolates were recovered from urine samples obtained from two patients admitted to a 4th level hospital in Bogotá – Colombia, in different months of 2015. GMR-RA1153 was isolated from a patient who was in an intensive care unit (ICU), and GMR-RA257 without data from the collection site. The two isolates were identified as *Providencia rettgeri*, using the automatic VITEK® 2 system (bioMérieux).

### Molecular identification

An amplification of the 16S rRNA gene (~ 1.5 kb) was made to confirm the genus and species of each isolate. For this, the universal primers Forward 27F 5 ‘AGAGTTTGATCMTGGCTCAG 3’ and Reverse 1492R 5 ‘TACGGYTACCTTGTTACGACTT 3’ were used. Purified PCR products were sequenced, using the BigDye methodology and following the manufacturer’s protocol (Applied Biosystems, Foster City, United States) at the sequencing service of the Institute of Genetics at Universidad Nacional de Colombia. Chromatograms were analyzed using Chromas program (Technelysium Pty Ltd.). The 16S rRNA sequencing data were aligned and compared with those available in the NCBI (National Center for Biotechnology Information) GenBank database using the BLASTn algorithm.

### Susceptibility testing

The antibiotic susceptibility profile was determined using the Kirby-Bauer disk diffusion susceptibility test. The following disks were tested: cefepime (FEP), cefoxitin (FOX), cefotaxime (CTX), ceftazidime (CAZ), ertapenem (ERT), imipenem (IMI), meropenem (MEM), aztreonam (ATM), ciprofloxacin (CIP), amikacin (AK), piperacillin/tazobactam (TZP) and trimethoprim/sulfamethoxazole (SXT). The susceptibility results were interpreted using the 2015 CLSI (Clinical and Laboratory Standards Institute) guidelines [50].

### Confirmation of carbapenemase production

Phenotypic detection of carbapenemases was performed using modified Hodge test (MHT) [33], and two double-disc synergy tests with specific inhibitors: ethylenediaminetetraacetic acid / mercaptoacetic acid (EDTA / SMA) and phenyl boronic acid (APB). For the MHT, the reference strain *Escherichia coli* ATCC 25922 was used as the indicator organism, strain *K. pneumoniae* BAA 1705 as positive control and strain *K. pneumoniae* BAA 1706 as negative control. For the EDTA / SMA test the positive control used was *K. pneumoniae* BAA 2146 and for the APB test was the strain *K. pneumoniae* BAA 1705. The synergy of the antibiotic imipenem towards EDTA/SMA was interpreted as a positive result for the presence of class B carbapenemase. Similarly, the halo distortion of the antibiotic imipenem towards APB was interpreted as a positive result for the presence of class A carbapenemase.

### Whole genome sequencing

The isolates’ genomic DNA was obtained by using two commercial kits, according to the manufacturer’s recommendations: QIAamp® DNA mini kit (Qiagen) for GMR-RA1153, and PureLink® Genomic DNA Kit (Thermo Fisher Scientific / Invitrogen) for GMR-RA257. DNA quantification was estimated by the PicoGreen method, using Vector 3 fluorometry. The final Paired-End sequencing was done with the Illumina HiSeq 2500 platform (the TruSeq Nano DNA kit (Illumina) was used for library construction). The quality of the raw paired-end reads was assessed by FastQC v0.11.7 [61], and the trimming of poor-quality bases (<Q30 and < 1000 bp) was done using the Sickle v1.33 tool [62]. Subsequently, the reads were de novo assembled using SPAdes v3.8.0 [39]. The annotation process was done using Prokka v1.13 [63], which was enriched with the following resistance databases: CARD [64], Resfam [65], GIPSy [66] and VFDB [67] and we complete the analysis with the Resfinder database (https://cge.cbs.dtu.dk/services/ResFinder/).

### *Providencia rettgeri* genome sequence data

The genomes GMR-RA257 and GMR-RA1153 were compared with 16 genomes of *Providencia rettgeri* available in GenBank: 14 reference genomes (RB151, H1736, CCBH11880, FDAARGOS_330, MR4, 729–12, UBA5024, AR_0082, TUM9994, Dmel1, DSM1131, PR1, Pret_2032, PR002); and 2 collection strains: (NCTC11801, NCTC74801). An important factor to take into account is that these genomes were used without discrimination of chromosome and plasmid sequences.

### Ribosomal multilocus sequence typing (rMLST)

The Ribosomal Multi-Locus Sequence Typing Scheme described by Jolley et al. [68] and hosted on the PubMLST website https://pubmlst.org/rmlst/ was used for the typing of the isolates of this work. Ribosomal Multilocus Sequence Typing (rMLST) is an approach that indexes the variation of the 53 genes encoding the bacterial ribosome protein subunits (rps, rpl, rpm genes) as a means of integrating microbial typing. The allelic variation rMLST is cataloged using the BIGSdb platform [44]: scalable, open source database that allows to store sequences and allelic definitions for defined loci [69] For the study, the rMLST scheme first identified the nucleotide sequences of the genes (rps, rpl, rpm) of the annotated genomes; then, defined the unique alleles for each of these genes based on their nucleotide sequence; and finally, assigned each genome a customized ribosomal sequence type (rST) number defined by the allelic profile of the numerical combination of the 53 genes.

### Whole genome comparisons

Using the *Providencia Rettgeri* genome sequence data described above the pangenome was inferred with Roary version 3.7.0 [70]. Roary produced a gene presence/absence matrix, a multi-FASTA alignment of core genes using PRANK version 0.140603 [71] and a phylogenetic tree based on the presence and absence of accessory genes among taxa using FastTree version 2.1.9 [72].

Visualization of circular genome BLASTN-based comparisons between Colombian reference genome RB151 and the genomes GMR-RA257 and GMR-RA1153, in order to show a broad genome visualization of the identity of the coding, was done using CGView Comparison Tool [73].

The average nucleotide identity (ANI) approach was used [74] to determine the genetic relationship between GMR-RA257 and GMR-RA1153. ANI was calculated within the JSpecies software [75]. GMR-RA257 and GMR-RA1153 were aligned with Mauve version 2.4.0 [76]. From this alignment, a file with the SNPs was obtained using Mauve 2.4.0 and converted to VCF format using an in-house Perl script. Finally, the SNPs were annotated with the snpEff program (http://snpeff.sourceforge.net).

The detection of these resistance genes for *Providencia Rettgeri* genome sequence data was performed using the program Resfinder (https://cge.cbs.dtu.dk/services/ResFinder/), using the fasta files of the 18 genomes of *P. rettgeri* (GMR-RA257 and GMR-RA1153, 14 reference genomes and 2 collection strains).

## Supplementary Information

**Additional file 1 Table S1.** Assembly and annotation Providencia rettgeri isolates GMR-RA257 and GMR-RA1153.

**Additional file 2 Table S2.** Antibiotic resistance present in GMR-RA257, GMR-RA1153, and 16 *Providencia rettgeri* genomes previously reported.

## Data Availability

Sequence data of this project have been deposited in the DDBJ/EMBL/GenBank of the National Center for Biotechnology Information (NCBI) under the accession number VRPG00000000 and VRPH00000000.

## Abbreviations

ANI: Average Nucleotide Identity
APB: Phenyl Boronic Acid
EDTA / SMA: Ethylenediaminetetraacetic acid / Mercaptoacetic acid
ICU: Intensive Care Unit
INS: National Institute of Health
MDR: Multi Drug-Resistant
MHT: Modified Hodge test
rMLST: Ribosomal Multilocus Sequence Typing
rST: Ribosomal Sequence Type
SNP: Single-Nucleotide Polymorphism
WGS: Whole-Genome Sequencing
