# Supplementary material for: Genome-based characterization of two Colombian clinical Providencia rettgeri isolates co-harboring NDM-1, VIM-2, and other β-lactamases
Source: BMC Microbiol. 2020 Nov 12;20:345. doi: 10.1186/s12866-020-02030-z (PMC7664025; doi:10.1186/s12866-020-02030-z)
Supplement: Supplementary file 1 — Additional file 1 Table S1. Assembly and annotation Providencia rettgeri isolates GMR-RA257 and GMR-RA1153. [file 12866_2020_2030_MOESM1_ESM.docx]

**Table S1. Assembly and annotation *Providencia rettgeri* isolates GMR-RA257 and GMR-RA1153**

|  | **GMR-RA257** | **GMR-RA1153** |
| --- | --- | --- |
| **Paired-end reads** | 20,137,464 | 5,928,353 |
| **Contigs > 1000pb** | 77 | 73 |
| **Genome coverage** | 744x | 216x |
| **Read**      **length (bp)** | 101 | 101 |
| **Total length of draft genome (bp)** | 4,836,247 | 4,838,047 |
| **GC content (%)** | 40,5 | 40,5 |
| **N50** | 187362 | 223809 |
| **Coding sequences (**     **CDS)** | 4452 | 4452 |
| **rRNAs** | 7 | 7 |
| **tRNAs** | 71 | 71 |
